# Pulmonary infiltrates during community acquired Gram-negative bacteremia: a retrospective single centre study

**DOI:** 10.1186/1757-7241-21-88

**Published:** 2013-12-17

**Authors:** Hans Fjeldsøe-Nielsen, Kirsten Gjeraa, Birgitte G Berthelsen, Ram B Dessau

**Affiliations:** 1 Department of Intensive Care, Odense University Hospital, Sdr. Boulevard 29, 5000 Odense C, Denmark; 2 Department of Intensive Care, Nykøbing Falster Hospital, Fjordvej 15, 4800 Nykøbing Falster, Denmark; 3 Department of Internal Medicine, Herlev Hospital, Herlev Ringvej 75, 2730 Herlev, Denmark; 4 Department of Clinical Microbiology, Slagelse Hospital, Ingemannsvej 18, 4200 Slagelse, Denmark

**Keywords:** Gram-negative bacteremia, Pulmonary infiltrates, Urosepsis, Lipopolysaccharide, Flagellin

## Abstract

**Background:**

The primary aim of this study was to describe the frequency of pulmonary infiltrates on chest X-ray (CXR) during community acquired Gram-negative bacteremia at a single centre in Denmark.

**Methods:**

The patients were retrospectively identified from the laboratory information system and clinical and radiological data were retrieved from the electronic health records. Overall 114 patients with *E.coli* or *K.pneumoniae* bacteremia fulfilled the inclusion criteria during the period 2009–2010.

**Results:**

CXR was performed in 77% of cases (80% of *E.coli* and 56% of *K.pneumoniae*) among which infiltrates were identified in 34%. The two most frequent localizations of infiltrates during *E.coli* bacteremia were lower lobe/basal (56%) and diffuse (22%). Furthermore, 30% of infiltrates were bilateral while 40% were present on the right lung and 30% on the left lung.

**Conclusions:**

In conclusion, the presence of infiltrates during community acquired Gram-negative bacteremia was very frequent in our population.

## Background

Patients admitted to hospital with sepsis and pulmonary infiltrates may have bacterial pneumonia, however, this is not always the case. The recommended treatment of community acquired pneumonia in Denmark is still in most cases penicillin taking into account disease severity and patient characteristics. The recommended treatment of urosepsis includes a broader spectrum beta-lactam antibiotic covering the most common Enterobacteriacae. It is well known that appropriate antibiotic treatment on admission reduces mortality during severe sepsis and septic shock. Therefore, the primary diagnosis and early focus identification is crucial in order to initiate appropriate empiric antibiotic treatment while waiting for microbiologic results. The primary purpose of this study was to determine how often pulmonary infiltrates are identified on chest X-ray (CXR) during community acquired Gram-negative bacteremia.

## Methods

### General information

Patients admitted to the emergency department at Nykoebing Falster Hospital with bacteremia with *E.coli* or *K.pneumoniae* during the period January 2009 to December 2010 were included in the study retrospectively. Patients with multiple bacteremic episodes were only included with the first episode fulfilling the inclusion criteria. Episodes with more than one isolated microorganism were excluded. Patients under the age of 14 years were excluded. CXR and microbiologic samples taken within 2 days of the positive blood culture were studied. CXR was considered positive by the authors if the radiologist’s reading described findings suggestive of acute infiltrate. Patients were defined as having a primary urinary tract focus when the urine and blood culture contained the same species. According to Danish law, approval of the Regional Ethics Committee was not required. The study was included in the simplified registration of Region Zealand at the Danish Data Protection Agency (Journal number 2008-58-0020).

### Statistics

The R statistical software was used. The 95% binomial confidence intervals on the binomial probabilities were calculated by the “Wilson” method (Command binom.confint, library binom, http://www.r-project.org).

## Results

During the 24 month period a total of 287 patients with 315 bacteremic episodes (excluding coagulase-negative Staphylococci and *Corynebacterium sp*.) were identified. Excluding duplicates 114 episodes fulfilled the inclusion criteria (Table 1). Median age was 75 (range from 27 years to 96 years) and 52% were male.

**Table 1:** Pneumonic infiltrates on chest radiography

**Bacteremia**	**BC**	**Chest radiography**	**+ infiltrate**	**- infiltrate**
*E.coli*	96	77 (80%, 71–87)	27 (35%, 25–46)	50 (65%, 54–75)
*K.pneumoniae*	18	11 (56%, 39–80)	3 (27%, 10–57)	8 (73%, 43–90)

*BC*, Blood culture.

N (%, 95% binomial confidence intervals).

CXR was available in 77% of bacteremic episodes (80% of *E.coli* and 56% of *K.pneumoniae*) (Table 1). Among these, infiltrates were identified in 34% (35% *E.coli* and 30% *K.pneumoniae*). Furthermore, CXR was performed in 44 (85%) of the 52 episodes of *E.coli* bacteremia with confirmed primary urinary tract focus. Among these an infiltrate was also present in 34% (15/44).

Localizations of infiltrates were only analysed for episodes of *E.coli* bacteremia as only three patients with *K.pneumoniae* had infiltrates. The two most frequent localizations of infiltrates among bacteremic episodes caused by *E.coli* were lower lobe/basal (56%) and diffuse (22%). Furthermore, 30% of infiltrates were bilateral while 40% were present on the right lung and 30% on the left lung.

Overall urine samples were obtained in 69% (*E.coli*) and 61% (*K.pneumoniae*) of bacteremic episodes. When available, urine cultures were positive with the same microorganism as the corresponding blood culture in 79% (*E.coli*) and 55% (*K.pneumoniae*) (Table 2).

**Table 2:** **Bacteremia with verified microbiologic focus, N (%, 95**% **binomial confidence intervals)**

**Bacteremia**	**BC**	**Urine positive**	**Respiratory positive**	**Other**
*E.coli*	96	52/66 (79%, 58–87)	0/2 (0%)	1
*K.pneumoniae*	18	6/11 (55%, 28–79)	0/0 (0%)	0

*BC*, Blood culture.

Lower respiratory tract samples were obtained in 2% (*E.coli*) and 0% (*K.pneumoniae*) of all episodes. Among patients with Gram-negative bacteremia none had a confirmed respiratory tract focus. Blood cultures were the single microbiological samples available in 32% of cases (31% of *E.coli* and 39% of *K.pneumoniae*). Furthermore, 33% of patients with pulmonary infiltrates who later on had confirmed *E.coli* bacteremia did not have urine samples collected.

## Discussion

To our knowledge articles addressing the presence of pulmonary infiltrates among patients with community acquired Gram-negative bacteremia have not previously been published. In this study 80% of all *E.coli* bacteremic episodes had CXR done upon admission verifying pulmonary infiltrates in 35%. Similar rates (27%) were found for *K.pneumoniae* but the limited number of data does not allow a certain estimate.

Antibiotic treatment for bacterial pneumonia in areas with low level of antimicrobial resistance is often not sufficient to treat other infections derived from e.g. the urinary tract or gastrointestinal tract. Therefore, the initial clinical diagnosis upon admission is crucial. This is especially the case in Scandinavia where the resistance of *S.pneumonia* to penicillin is still low.

A study from the County of Northern Jutland, Denmark showed that among 815 episodes of monomibrobial Gram-negative bacteremia (70% *E.coli*, 17% *K.pneumonia*) 128 (16%) did not receive any antibiotic treatment before the notification of a positive blood culture and 94 (12%) were treated with penicillin G. The presence of pulmonary infiltrates was not investigated in that study [1].

Only a small proportion of patients (2%) in this study had both respiratory tract and urine cultures collected, thus identification of the primary focus had to be based on the samples available. Ideally both samples should be collected, without unnecessary delay, before initiation of antibiotic treatment in order to confirm the primary focus. Surprisingly, 31% of *E.coli* and 39% of *K.pneumoniae* bacteremic episodes did not have any supplementary microbiological samples collected apart from blood cultures.

It is well known that Gram-negative sepsis may cause acute lung injury (ALI) or acute respiratory distress syndrome (ARDS) [2]. A possible immunological explanation could be LPS (lipopolysaccharide) induced neutrophil recruitment to the lungs. LPS has been identified as a reliable indicator of Gram-negative bacteremia comparable to blood cultures [3] but whether LPS concentrations are correlated with the presence of pulmonary infiltrates has not been studied.

In vivo studies have shown that LPS activates macrophages and epithelial cells regardless of the route of administration [4,5]. This triggers a chemotactic inflammatory response with production of proinflammatory cytokines and oxygen metabolites which causes an influx of neutrophils into the airways. Platelets adhere to vascular wall causing endothelial damage and increased permeability [4-6]. Reactive oxidants may lead to ARDS which is caused by an imbalance between oxidants and endogenous antioxidants [7,8]. The immunologic response to LPS exposure from different Enterobacteriacea is not the same which has recently been shown to have prognostic value [9].

Another possible pathophysiological explanation for the inflammatory cascades in the lungs after exposure to Gram-negative bacteria is mediated by flagellin, a component of bacterial flagella. In a small sample of patients (N = 16) flagellin level was correlated to lung injury score, alveolar-arterial oxygen difference and duration of septic shock. Furthermore, flagellin was found to be a more potent stimulus than LPS for the release of proinflammatory cytokines in the lungs of mice [10].

A weakness of this study is that patient characteristics were not analyzed in relation to primary clinical diagnosis, co-morbidity or severity scores. Furthermore, microbiological samples were not systematically collected. These parameters should be included in future studies. However, the primary purpose of our study has been achieved showing that overall pulmonary infiltrates on CXR, whatever the cause, are very frequent among patients admitted with Gram-negative community acquired bacteremia in our population.

## Conclusions

This study has shown that pulmonary infiltrates are common among patients with community acquired Gram-negative bacteremia in Denmark. The study also showed that only a small number of our patients had both urine and respiratory tract samples collected. Emergency room physicians should have this in mind when treating patients with community acquired sepsis as focus identification and initial diagnosis is important to initiate effective antimicrobial therapy as early as possible. These findings could have an important impact on initial patient assessment in the emergency department if supported by future studies.

## Competing interests

The authors declare that they have no competing interest.

## Authors’ contributions

HFN contributes collecting and analyzing data and writing the manuscript. KG contributes collecting data and commenting the manuscript. BGB contributes analyzing the data and commenting the manuscript. RBD contributes with statistical analysis and commenting the manuscript. All authors read and approved the final manuscript.

## Authors’ information

Both HFN and RBD are medical specialists in Medical Microbiology. The idea of this article originated after years of consulting clinicians on optimal antibiotic treatment and focus identification of septic patients with documented bacteremia. The primary clinical diagnosis was often found to be pneumonia even when microbiological samples clearly indicated bacteremia originating from the urinary tract. This initial discrepancy is well known among medical microbiologists in Denmark even though no articles have yet been published on the subject.

## References

[B1] Pedersen G Schønheider HC Sørensen HT Antibiotic therapy and outcome of monomicrobial gram-negative bacteremia: a 3-year population-based study Scand J Infect Dis 1997 21 601 606 10.3109/00365549709035903 9571742

[B2] Kollef MH Schuster DP The acute respiratory distress syndrome N Engl J Med 1995 21 27 37 10.1056/NEJM199501053320106 7646623

[B3] van Deventer SJ Buller HR ten Cate JW Sturk A Pauw W Endotoxaemia: an early predictor of septicaemia in febrile patients Lancet 1988 21 605 609 2894546 10.1016/s0140-6736(88)91412-2

[B4] Brigham KL Meyrick B Endotoxin and lung injury Am Rev Respir Dis 1986 21 913 927 3085564

[B5] Tauseef M Knezevic N Chava KR Smith M Sukriti S Gianaris N TLR4 activation of TRPC6-dependent calcium signaling mediates endotoxin-induced lung vascular permeability and inflammation J Exp Med 2012 21 1953 1968 10.1084/jem.20111355 23045603 PMC3478927

[B6] Reiss LK Uhlig U Uhlig S Models and mechanisms of acute lung injury caused by direct insults Eur J Cell Biol 2012 21 590 601 10.1016/j.ejcb.2011.11.004 22284832

[B7] Chen H Bai C Wang X The value of the lipopolysaccharide-induced acute lung injury model in respiratory medicine Expert Rev Respir Med 2010 21 773 783 10.1586/ers.10.71 21128752

[B8] Sabarirajan J Vijayaraj P Nachiappan V Induction of acute respiratory distress syndrome in rats by lipopolysaccharide and its effect on oxidative stress and antioxidant status in lung Indian J Biochem Biophys 2010 21 278 284 21280564

[B9] Hurley JC Opal SM Prognostic value of endotoxemia in patients with Gram-negative bacteremia is bacterial species dependent J Innate Immun 2013 [Epub ahead of print] 10.1159/000347172 PMC6741488 23594418

[B10] Liaudet L Szabó C Evgenov OV Murthy KG Pacher P Virág L Flagellin from gram-negative bacteria is a potent mediator of acute pulmonary inflammation in sepsis Shock 2003 21 131 137 10.1097/00024382-200302000-00008 12578121

